# Barriers to viral suppression in children aged 9 years or younger on dolutegravir-based antiretroviral therapy in Malawi, a mixed-methods study

**DOI:** 10.1371/journal.pgph.0004510

**Published:** 2025-05-30

**Authors:** Khumbo Phiri, Christine Hagstrom, Eric Lungu, Kelvin Balakasi, John Songo, Amos Makwaya, Deanna Smith, Anteneh Worku, Risa Hoffman, Sam Phiri, Kathryn Dovel, Joep J. van Oosterhout

**Affiliations:** 1 Partners in Hope, Lilongwe, Malawi; 2 Department of Medicine, David Geffen School of Medicine, University of California, Los Angeles, California, United States of America; 3 Department of Health, Nutrition and Population, USAID Malawi, Lilongwe, Malawi; 4 School of Global and Public Health, Kamuzu University of Health Sciences, Lilongwe, Malawi; Makerere University, UGANDA

## Abstract

After the transition to pediatric dolutegravir-based regimens, viral load (VL) suppression among children with HIV (CWH) in Malawi has remained suboptimal. This mixed-methods study assessed factors associated with high VL among young CWH on dolutegravir-based antiretroviral therapy (ART) and explored adherence barriers from primary-caregiver and healthcare worker perspectives. Between April-July 2023, we performed an unmatched case-control study at 49 Malawian health facilities. We included CWH aged ≤9 years, on dolutegravir-based ART, with a routine VL test-result that was high (≥1,000 copies/mL) for cases, or suppressed (<200 copies/mL) for controls. Using mixed-effect modified Poisson regression, we determined factors associated with high VL, adjusting for sex, site and district. To assess adherence barriers, we conducted in-depth interviews (IDIs) with randomly selected caregivers of CWH with high VL and with healthcare workers providing pediatric HIV care. Data were analyzed using a hybrid thematic approach that combined deductive and inductive coding strategies. We enrolled 538 CWH: 222 cases, with high VL and 316 controls, with suppressed VL. Duration on ART > 4 years (aRR = 0.86, 95% CI: 0.77-0.95) and ≥2 interruption in treatment episodes (≥28 days late for clinic appointment) in the 12 months before VL sample collection (aRR = 1.47, 95% CI: 1.28-1.68) were significantly associated with high VL. Through 54 IDIs (30-caregivers, 24-healthcare workers), five key adherence challenges were identified and affected the children ≤5 years the most: resistance to daily medication, difficulties taking multiple pills, food insecurity, fear of unintentional disclosure, and inability to attend clinic appointments consistently. This study highlights that behavioral, socio-economic and psychosocial factors influences ART adherence among CWH. Duration on ART and recent interruptions in treatment were associated with high VL, stressing the need for targeted interventions that will require health-system and client-level approaches to improve VL suppression among CWH in Malawi and similar settings.

## Introduction

The UNAIDS 2025 targets include 95% of people with HIV (PWH) know their status, 95% of those diagnosed receive treatment, and 95% of those on treatment achieve viral suppression. However, in eastern and southern Africa, children with HIV (CWH) aged under 14 years have much higher rates of viremia compared to adults and are not close to reaching the UNAIDS “third 95” [1]. Achieving and maintaining viral suppression is crucial for CWH to prevent disease progression, reduce morbidity and mortality, and improve overall health outcomes. Dolutegravir’s high genetic barrier to drug resistance and its good tolerability [2] have resulted in high rates of viral suppression among adults on dolutegravir-based ART [3–6]. Consequently, the World Health Organization (WHO) recommends using dolutegravir-based ART as a first-line and second-line treatment [7]. The introduction of pediatric dolutegravir formulations has enhanced ART care for CWH by improving acceptability and ease of administration of antiretroviral medications in this age group [8].

Prior to the dolutegravir transition, viral load suppression rates among children living with HIV in Malawi, Zimbabwe, and Uganda were poor, with only 65–69% achieving viral suppression between 2016–2018 [9]. Malawi started transitioning children to dolutegravir-based regimens in 2019. Since then, national HIV program data indicate that despite some improvement, around 20% of CWH have high viral load (VL) despite being on dolutegravir-based regimens, with children aged under 4 years having the worst VL suppression outcomes [10]. In CWH on dolutegravir-based ART, viremia is expected to be related mostly to non-adherence and only occasionally to dolutegravir resistance [11]. However, limited information is available from the region about factors contributing to high VL in the dolutegravir era**.** Failure to fully understand causes of high VL among children on dolutegravir-based regimens risks continued poor treatment outcomes, drug resistance and increased morbidity and mortality. Comprehensive investigation of these factors is essential, as understanding the root causes will enable the development of targeted interventions to improve clinical outcomes, preserve treatment options, and ultimately reduce HIV-related illness and death in this vulnerable population

We conducted a mixed methods study to assess factors associated with high VL among CWH on dolutegravir-based ART in Malawi and explored barriers to CWH’s adherence from the perspective of primary-caregivers (caregivers) and healthcare workers (HCWs). Our study focused on children aged ≤9 as the adherence challenges faced by this demographic are distinct from those of adolescents, who begin transitioning toward medication self-management and face a different set of barriers beyond caregiver-dependent administration.

## Methods

### Study setting

This study was conducted at 49 health facilities supported by Partners in Hope (PIH), a Malawian non-governmental organization serving as a PEPFAR/USAID implementing partner for HIV care and treatment until February 2025. The facilities included 26 government health centers, 12 government district and rural hospitals, and 11 faith-based hospitals, distributed across nine districts (Nsanje, Chikwawa, Mulanje, Lilongwe, Nkhotakota, Kasungu, Dowa, Karonga and Chitipa), spanning all three regions of Malawi. PIH supported HIV care and treatment for approximately 2,098 CWH ≤9 years across the 49 study sites, including a comprehensive approach to managing high VL results. This encompassed intensive clinical monitoring, enhanced adherence counseling, psychosocial support for guardians, support for disclosure of the HIV status, management of comorbidities, linkage to orphan and vulnerable children (OVC) services, and HIV drug resistance testing, when indicated.

### Study design

We employed a mixed method approach to assess factors associated with high VL among CWH aged ≤9 years. Our study focused on children ≤9 years, as viremia is most prominent in this age group and because among teens, factors associated with high VL and poor adherence may be substantially different, as they start taking ARV medication independently from caregivers.

The quantitative component utilized an unmatched case-control study design. We defined cases as CWH aged ≤9 years who had routinely collected high VL test results, (≥1,000 copies/mL) between January-December 2022. Controls were CWH aged ≤9 years whose routinely collected VL test results were suppressed (<200 copies/mL) within the same time period. We retrospectively collected data on clinical and treatment factors 12-months prior to the VL sample collection date. We then conducted in-depth interviews with caregivers of enrolled CWH who had high VL and with HCWs who provide pediatric HIV care at the participating health facilities.

### Participant selection

#### Quantitative analysis.

Children were eligible for inclusion if they were 1) receiving HIV care at a participating health facility for at least one year; 2) aged ≤9 years; 3) on dolutegravir-based ART, irrespective of duration; and 4) had a routinely collected VL test result between January-December 2022 that was either high for cases (defined as ≥1,000 copies/mL) or suppressed for controls (defined as <200 copies/mL). CWH with VL results between 200–999 copies/mL were excluded because the virologic failure definition in Malawi is based on the ≥ 1,000 copies/mL threshold, which triggers intensive management, whereas the approach to management of 200–999 copies/mL VL results has varied over time.

Two-stage sampling was used to select participants. First, all children aged ≤9 years with high VL results recorded in standardized VL registers at the selected health facilities were selected for inclusion. Second, a systematic random sampling strategy was employed for children with suppressed VL, selecting every third child aged ≤9 years with a suppressed VL result recorded in the same VL registers. The sample size was determined by the number of children with high VL at the selected health facilities within the study period. Our final sample achieved 80% power at the 95% confidence level to detect a difference of 10% in adherence to routine clinic visits (a measure of treatment interruption) between cases and controls [9]. This 10% difference was deemed appropriate given that studies in similar settings have reported adherence to clinic visit differences ranging from 8% to 12% between children with high and suppressed VL [12–14].

#### In-depth interviews.

Sample size was determined based on qualitative research principles suggesting thematic saturation typically occurs within 20–30 interviews in relatively homogeneous populations [15]. We randomly selected 30 children with high VL, stratified by sex (15 males, 15 females). Caregiver and HCW IDIs were conducted at the seven health facilities with the largest number of children with high VL results. Research assistants (RAs) contacted primary caregivers (individuals primarily responsible for the child’s daily care, including medication administration and clinic attendance) of selected children by telephone and invited them to the health facility to participate in a one-time IDI. When caregivers declined to participate, or they could not be reached, random selection continued until we had completed the target sample size (n = 30). For HCW IDIs, we used purposive sampling to selected 24 HCWs who provided pediatric HIV care in the past three months or longer. Cadres included clinical staff (clinical officers, medical assistants, and nurses) and lay cadres who support HIV treatment, counseling, health promotion and community-based care (i.e., HIV Diagnostic Assistants (HDA), Treatment Supporters (TS), and Psycho-social Counsellors [PSC]). The selection of healthcare staff was based on having equal representation across different cadres.

### Data collection

#### Quantitative analysis.

Between April and June 2023, we collected individual-level data for every clinic visit recorded in the 12 months preceding the viral load sample collection date. Data were collected using routinely collected electronic monitoring systems and individual treatment charts. Variables of interest included: demographics, baseline ART clinic data, clinic appointment details, and ART regimen prior to VL sample collection. Study variables were measured as follows: demographic characteristics included age at VL sample collection time (calculated from date of birth to date prior to sample collection time for the last VL), sex, and district of residence. Baseline clinical characteristics comprised age at ART initiation (calculated from date of birth to ART start date) and duration on ART (calculated from ART start date to viral load sample collection date). Interruption in treatment (IIT) in the prior 12 months before VL sample collection was defined as ≥28 days late for a scheduled visit.

Data were digitized using Survey CTO software (Dobility Inc., Cambridge, Massachusetts, USA) on electronic tablets.

#### In-depth interviews.

Semi-structured interview guides were developed using existing literature about barriers to ART retention and adherence, specifically for children [9,16], and the social ecological framework to examine factors associated with viral load across individual, interpersonal, community and facility levels [17]. This multi-level framework directed the development of the interview guide, which included the following domains: children’s lived experiences; family dynamics; caregiver relationships; social context that influence decisions to adhere to HIV treatment; and specific barriers to adherence to ART reported by respondents. Trained RAs conducted all interviews between June 8^th^ and July 13^th^ 2023. Interviews took place in private spaces at the health facilities to ensure confidentiality. Prior to each interview, RAs obtained written informed consent from participants. All interviews were conducted in the local language (Chichewa) and were audio recorded, transcribed verbatim and translated to English.

### Statistical analysis

#### Quantitative analysis.

The characteristics of children with high versus suppressed VL were compared using descriptive statistics. Using univariate regression analyses, we explored associations between demographic, baseline clinical, and clinic visit characteristics with high VL. In regression analysis, we utilized mixed-effect modified Poisson regression modeling because the outcome was common (>10%) and to account for clustering at the district and site levels. In multivariable regression, we included all factors that were significant from the univariate regression (p < 0.1), adjusting for sex, site and district. Multicollinearity among the covariates was checked, and all analyses were performed using Stata 18 (StataCorp LLC, Texas, USA).

#### In-depth interviews.

Transcripts were analyzed in Atlas.ti version 23 software (Atlas.ti Scientific Software Development GmbH, Germany). We employed a hybrid thematic analysis approach, combining both deductive and inductive coding strategies. Initial deductive codes were developed based on existing literature on pediatric HIV treatment adherence and the social ecological framework. This theoretical foundation guided our preliminary coding structure while still allowing for emergent themes through inductive coding. A codebook was developed and finalized after the first five transcripts were coded. Independent coding was completed by three authors (KP, EL and CH). Throughout the analysis, we focused on dominant overarching themes. Additionally, we employed constant comparison methods to examine how themes manifested differently between the two age groups (≤4 years versus >4–9 years) and between caregivers and HCWs. This comparative approach allowed us to identify age-specific patterns in treatment adherence barriers and experiences.

### Ethical statement

The study was conducted in compliance with ethical guidelines as follows: 1) the overall protocol was approved by the Malawi National Health Sciences Research Committee (protocol 23/03/4022) and the University of California, Los Angeles (IRB number 23–000686); 2) The quantitative phase on medical records, using routine program data, was conducted under protocol #1099, approved by the National Health Sciences Research Committee of Malawi for program evaluation activities. For IDIs, written informed consent was obtained from participants, who received the equivalent of ten dollars compensation for their participation.

## Results

### Quantitative analysis

We included 538 children in the medical charts analysis, predominantly aged 5–9 years at VL sampling (87.7%, n = 472) with a small proportion aged 4 years and younger (12.3%, n = 66). The sample contained slightly more females (52.6%, n = 283) than males (47.4%, n = 255). More than 99% were on abacavir/lamivudine with dolutegravir, 0.4% on zidovudine/lamivudine with dolutegravir and 0.4% on tenofovir DF/lamivudine/dolutegravir. The proportion with high VL was 41.3% (n = 222) and 58.3% (n = 316) had suppressed VL (Table 1). More children aged ≤4 years at VL sample collection time had high VL (53.0%) than those aged 5–9 years (39.6%). High VL was also more common among children who had been on ART for ≤4 years (47.0%) than among those on ART for ≥5 years (38.2%). The median duration of IITs was 42 days (IQR 32–62 days). IIT in the last 12 months occurred more frequently among children with high VL compared to those with suppressed VL, and children with high VL were more likely to have two or more IIT episodes compared to those with suppressed VL (9.0% versus 3.2%). Other variables, such as sex, age at ART initiation, duration on current ART regimen, district, and ART dispensing interval, did not differ between the two groups.

**Table 1:** Characteristics of children included in medical chart review (n = 538).

Variable	Total	Cases, Children with high VL (≥1,000 copies/mL)	Controls, Children with suppressed VL (<200 copies/mL)
% (N)	100% (538)	41.3% (222)	58.7% (316)
*Sex, % (N)*			
Female	52.6 (283)	48.6 (108)	55.4 (175)
Male	47.4 (255)	55.4 (175)	48.6 (108)
*Median age at ART initiation (IQR)*	1.6 (1.0-2.6)	1.7 (1.0-2.7)	1.6 (1.0-2.5)
*Age at ART initiation, % (N)*			
≤ 4 years old	88.3 (475)	87.4 (194)	88.9 (281)
≥ 5–9 years old	11.7 (63)	12.6 (28)	11.1 (35)
*Duration on current DTG in prior 12 months of viral load, % (N)*			
0–6 months	12.8 (69)	13.1 (29)	12.7 (40)
> 6 months	87.2 (469)	86.9 (193)	87.3 (276)
*Median age at time of VL sample collection (IQR)*	6.7 (4.9-8.1)	6.6 (4.6-8.3)	6.7 (5.1-8.0)
*Age at VL sample collection, % (N)*			
≤ 4 years old	12.3 (66)	15.8 (35)	9.8 (31)
≥ 5–9 years old	87.7 (472)	84.2 (187)	90.2 (285)
*Median years on ART, (IQR)*	5.0 (3.5-6.5)	4.7 (3.1-6.6)	5.2 (3.7-6.5)
*Years on ART, % (N)*			
≤ 4 years	34.4 (185)	39.2 (87)	31 (98)
≥ 5–9 years	65.6 (353)	60.8 (135)	69 (218)
*ART dispensing interval of the visit prior to VL sample collection, % (N)*			
1 month	64.1 (345)	67.6 (150)	61.7 (195)
2–3 months	31.4 (169)	28.4 (63)	33.5 (106)
> 3-months	4.5 (24)	4.0 (9)	4.8 (15)
*IIT in the past 12-months, % (N)*			
None	374 (69.5)	142 (64.0)	232 (73.4)
One IIT episode	134 (24.9)	60 (27.0)	74 (23.4)
Two or more IIT episodes	30 (5.6)	20 (9.0)	10 (3.2)
*Among those with IIT (n = 164)*			
*Median IIT days, (IQR)*	42.0 (32-62)	42.0 (32-42)	39.5 (32-63)
*Median number of IIT episodes, (IQR)*	1 (1-2)	1 (1-2)	1 (1-1)
*District, % (N)*			
Lilongwe	21.4 (115)	19.4 (43)	22.8 (72)
Kasungu	5.6 (30)	6.3 (14)	5.1 (16)
Karonga	7.1 (38)	7.2 (16)	6.9 (22)
Mulanje	12.3 (66)	12.2 (27)	12.3 (39)
Nsanje	16.2 (87)	18.0 (40)	14.9 (47)
Chikwawa	28.8 (155)	26.6 (59)	30.4 (96)
Nkhotakota	6.3 (34)	7.7 (17)	5.4 (17)
Dowa	2.4 (13)	2.7 (6)	2.2 (7)

IQR, interquartile range; DTG, dolutegravir; VL, viral load; ART, antiretroviral therapy; IIT, interruption in treatment.

### Factors associated with high viral load

Duration on ART was independently associated with high VL (≥1,000 copies/mL). Children who had been on ART longer than 4 years had a lower risk of high VL (aRR 0.86; 95% CI: 0.77-0.95) compared to those who had been on ART for 4 years or less. Children who experienced two or more IIT episodes in the 12 months prior to VL sampling had an increased risk of high VL (aRR 1.47; 95% CI: 1.28-1.68) compared to those having no IIT episodes. No other variables were independently associated with high VL (Table 2).

**Table 2:** Factors associated with high viral load (n = 538).

Variable	RR	95% CI	aRR^	95% CI
*Sex*				
Male	Ref			
Female	0.89	0.80-1.01	–	–
*Age at ART initiation*				
0–4 years old	Ref			
5–9 years old	1.06	0.87-1.30	–	–
*Duration on DTG regimen*				
0–6 months	Ref			
> 6 months	0.99	0.86-1.12	–	–
*Age at VL sample collection*				
5–9 years old	Ref			
0–4 years old	1.22	0.89-1.68		
*Years on ART*				
≤ 4 years	Ref			
> 4 years	0.86	0.78-0.96*	0.86	0.77-0.95**
*Last ART dispensing interval*				
1 month	Ref			
2–3 months	0.77	0.53-1.13		
> 3 months	0.78	0.33-1.83	–	–
*IIT in the12-months prior to VL sample collection*				
None	Ref		Ref	
One IIT episode	1.12	0.94-1.35	1.12	0.92-1.37
≥ 2 IIT episodes	1.45	1.28-1.65***	1.47	1.28-1.68***

^Adjusting for district, site, and sex.

*p < 0.1; **p < 0.05; ***p < 0.01.

RR, Risk ratio; aRR, adjusted risk ratio; CI, confidence interval; Ref, reference; DTG, dolutegravir; VL, viral load; ART, antiretroviral therapy; IIT, interruption in treatment.

### In-depth interview participants

We interviewed 30 caregivers of children with high VL (Table 3). Most caregivers were biological mothers (90%, n = 27), female (97%, n = 29), aged over 35 years (57%, n = 17), living with HIV and on ART (93%, n = 28), and had been on ART for more than 5 years (57%, n = 16). Most had only completed primary education (67%, n = 20) and were engaged in small business operations or trade (50%, n = 15). The CWH for whom they were caregivers, were evenly distributed by gender (50% female, n = 15) with a median age of 5 years (IQR: 5–8 years). The majority of children had initiated ART during infancy, immediately after diagnosis (70%, n = 21). The children’s median duration on ART was 4 years (IQR: 3–6 years). Less than half of caregivers (40%, n = 12) reported that the child knew their own HIV status. HCWs (n = 24) were predominantly female (71%, n = 17), aged between 18–35 years (50%, n = 12), and had attained tertiary-level training (67%, n = 16). The sample included an equal distribution of Clinical Officers/Medical Assistants, HIV Diagnostic Assistants, Nurses, and Treatment Supporters/Psychosocial Counsellors (25%, n = 6 each). Almost all participants (96%, n = 23) had worked in their positions for more than one year. Most HCWs were involved in clinical services (49%, n = 18) and counselling services (41%, n = 15), while a smaller proportion provided health promotion services (10%, n = 4). Some HCWs provided multiple types of services, as indicated by the total service count exceeding the number of participants.

**Table 3:** Characteristics of caregivers, their children, and HCWs participating in IDIs (n = 54).

Characteristic	Caregivers 30 (100%)	Children of participating caregivers 30 (100%)
*Age*		
< 5 years	0	15 (50%)
18–35 years	13 (43%)	0
> 35 years	17 (57%)	0
Not indicated	0	0
*Sex*		
Female	29 (97%)	15 (50%)
*Caregiver relationship to child*		
Biological father	1 (3%)	*N/A*
Biological mother	27 (90%)	*N/A*
Other	2 (7%)	*N/A*
*On ART*		
Yes	28 (93%)	*N/A*
*Duration on ART*		
< 5years	12 (43%)	16 (53%)
> 5years	16 (57%)	14 (47%)
*Time to initiate ART after diagnosis*		
Delayed	*N/A*	7 (23%)
Immediate	*N/A*	21 (70%)
Not indicated	*N/A*	2 (7%)
*Schooling*		
Attending school	0	7 (23%)
No school	2 (7%)	23 (77%)
Primary level	20 (67%)	0
Secondary	7 (23%)	0
Tertiary	0	0
Not indicated	1 (3%)	0
*Occupation*		
Business	15 (50%)	*N/A*
Farming	4 (13%)	*N/A*
Fulltime job	3 (10%)	*N/A*
Peace work	7 (23%)	*N/A*
Not indicated	1 (3%)	*N/A*
*HIV Status disclosed to child*		
Yes	*N/A*	12 (40%)
No	*N/A*	18 (60%)

N/A, not applicable; ART, antiretroviral therapy; IDI, in-depth interview.

### In-depth interview results

Our findings from the IDIs with caregivers and HCWs caring for CWH showed five key barriers to pediatric ART adherence that we describe in detail below.

#### Children resisting daily medication.

Children resisting or refusing to take medication was frequently mentioned as an important barrier to consistent medication adherence by both caregivers (13/30) and HCWs (10/24). As one caregiver explained:

*“Sometimes she would bluntly refuse them [medication]. Saying, “I don’t want them.” Then she would cry. So, I have to force, and plead with her to take them.” (Caregiver of a 5-year-old child, Chikwawa district).*

Several reasons for children’s resistance were mentioned, including medication side effects (e.g., skin rashes, diarrhea, vomiting, dizziness), bitter taste and unpleasant smell of the pills, defiant behavior, and treatment fatigue.,

*“She says whenever she takes them [medications] she experiences some heart problems. She fails to play.” (Caregiver of a 9-year-old child, Mulanje district).*

#### Difficulties in managing to take multiple pills at once.

Several caregivers (11/15) and HCWs (10/24) pointed to the number and size of pills children are required to take in a single day as an adherence barrier, particularly for children who were ≤4 years old. Some caregivers mentioned that they would prefer just one pill (instead of multiple) or injectables to decrease pill burden for their children. One mother explained:

*There are white ones, he takes 2 tablets and a half, then the tiny pink tablets, he takes 2 of them, Bactrim, he takes 2 of them. And I explained to hospital staff about how he complains about having to take too many sweets [pills] every day...according to me and my child, the government should take a stand on the injectable drugs because he will only need those once for a period of time.“* (Caregiver of a 5-year-old child, Lilongwe district).

HCWs also highlighted the scope of the issue:

*“They [the pills] are numerous...Let’s say a child is taking the drugs from this bottle, abacavir and lamivudine and he is taking 3, then he is taking 3 of dolutegravir, thus 6, he is taking 2 Bactrim tablets, that is 7 and 8 and then we are also giving TB prophylaxis, so number 9 or 10. There are 10 tablets for a child to take, it’s a challenge. It’s hard.”* (Clinician, Lilongwe district).

#### Lack of food.

Lack of food was frequently cited by caregivers (17/30) as hindering medication adherence. Caregivers expressed concerns about increased side effects and the safety of administering potent medication to children without food. One caregiver explained:

*“The challenge is shortage of food and when there is no food it becomes difficult to give medications to a child since these medications are strong.”* (Caregiver, Lilongwe district).

Many caregivers explained that food was an effective incentive to persuade children to take their medication, and without it, children were more likely to refuse and miss doses entirely. Notably, the use of incentives was common across all age categories with food for younger children and money used in older children.

*I tell him to take his medication then I should pour him some porridge, or like I said I bribe* (Caregiver of a 7-year-old child, Chikwawa district).

Another caregiver mentioned:

*When am feeding her porridge, 2 pills are already in the cup. Then after some time, I will give her another 2 pills then after some time the remaining 2 then am done for the day. When there is no food, it becomes difficult to give medications to a child* (Caregiver of a 3-year-old child, Lilongwe district).

HCWs echoed these concerns noting that children’s resistance to take medication combined with caregivers’ beliefs that medication should only be taken with food (even though this is not the required for medical reasons) often resulted in missed doses. A nurse explained:

*“What we have seen is that most children fail to adhere because of being poor in terms of food [food insecurity]. Most parents believe that medicine is only supposed to be taken after having a meal so due to the starvation going around in homes, they don’t give the child medicine.”* (Nurse, Chikwawa district).

Caregivers said they often could not give children their medicine because they struggled to provide basic needs like food, highlighting how unstable living conditions directly impact health management decisions. A Caregiver explained:

*I can’t give the child meds when we don’t have food. You should understand me when I say that I am poor, because really, I am poor. Because I just can’t give the child food on an empty stomach, he would get weak as a result.* (Caregiver of 4-year-old child, Lilongwe district).

Both caregivers and HCWs emphasized the link between food insecurity and poor pediatric adherence, with some suggesting that providing food assistance to families could improve adherence.

#### HIV status disclosure challenges and consequences.

Caregivers expressed concerns about disclosure and a majority (18/30) had not disclosed the HIV status to the child, with none of the children below 5 years reportedly having started disclosure, citing child’s young age as a primary reason. Most caregivers mentioned that the age of 10 years is appropriate to disclose the HIV status to their children. One mother explained:

*My child is very intelligent and asks me a lot of questions like ‘when will I stop taking the drugs?’ I could lie to him saying when you finish these ones, we will not get more meds at the hospital but we would get more meds and he refuses to take them sometimes. He is young and I can’t tell him the truth* (Caregiver of 5-year-old child, Mulanje district).

Caregivers expressed worries about the consequences of disclosure, fearing children may unintentionally disclose their status to others, and consequently face stigma. One caregiver said:

*“The problem is that children always tell one another that my mother has told me I am HIV positive and children start telling each other and then everyone becomes aware that the child is HIV positive.”* (Caregiver of a 4-year-old child, Mulanje district).

Most HCWs (13/24) identified multiple reasons for caregivers’ reluctance to disclose, extending beyond unintentional disclosure:

*“Women say are afraid the child will kill themselves if they disclose to them or that their marriage will end because they remarried after their first marriage, so the woman does not want to disclose that the child is on ART.”* (Clinician, Chikwawa district).

HCWs observed that non-disclosure often led to treatment adherence challenges. They noted that caregivers’ attempts to mislead children about their medication purpose and its (life-long) duration created confusion and frustration among children who questioned why they, unlike their peers, required daily medication.

*Other children do not know why they are taking drugs. They even go as far asking why their friends do not take drugs. Their parents lie to them saying they are taking drugs so that they cure the headache they have. So, whenever they feel no headache, they do not take their ART* (HDA, Lilongwe district).

Despite acknowledging these concerns, many HCWs emphasized the importance of disclosure in improving adherence, particularly when children are old enough, citing 10 years and above as an ideal age for children to understand. One HCW explained:

*“The advantage is that it improves adherence. It is like you are giving the child a responsibility. After disclosing to them, the child becomes aware why they are taking drugs and also the consequences of not taking them consistently. They become aware they are taking drugs to make sure their body soldiers become strong and able to defeat diseases.”* (Medical Assistant, Lilongwe district).

#### Missed clinic appointments resulting in treatment interruptions.

The majority of caregivers (22/30) reported struggling to consistently attend clinic appointments for ART refills for their children.

*“Sometimes maybe her appointment date comes and I am not home. So, we miss that appointment date and come on another day.”* (Caregiver of an 8-year-old child, Chikwawa district).

Caregivers cited several reasons for missing clinic appointments including challenges with transport, such as cost and accessibility, illness preventing them from attending appointments with their child, competing priorities in daily life such as work or travel obligations, and children being in school during clinic hours. One caregiver described how this can result in prolonged gaps in medication:

*“Not having any means of transportation. For instance, during the time of floods, I stayed for a month without going to get medication.”* (Caregiver of a 7-year-old child, Chikwawa district).

Competing priorities for both caregivers and older children could cause them to miss appointments:

*“I happen to be occupied with something or I travel out and the dates for us to go the hospital are due while am not home. I was at a certain place, this makes him not to come here on his dates.”* (Caregiver of a 5-year-old child, Lilongwe district).

## Discussion

In this mixed methods study, we found high VL to be associated with duration on ART of >4 years and two or more IITs in the 12 months before VL sample collection. Insights from caregivers and HCWs involved in pediatric HIV services pointed to five key adherence challenges: children’s resistance to take daily tablets; pill burden; food insecurity; challenges and consequences of HIV status non-disclosure; and missing clinic appointments. These findings underline the complexity and multitude of behavioral, psychosocial and socio-economic factors influencing adherence in young children in this setting.

The protective effect of longer ART duration observed in our study, with children on ART longer than 4 years having a 14% lower risk of high VL, aligns with regional evidence. Studies from Ghana, Ethiopia and Mozambique demonstrated similar patterns, with shorter ART duration (1–4 years) associated with increased risks of high VL [18–20]. This association likely stems from guardians and children gaining valuable experience with ART over time, as they may gradually become more familiar with medication routines through sustained engagement with healthcare services [12]. Supported by their caregivers, young children may develop more consistent medication-taking habits as they grow from infancy through early childhood. These findings emphasize the importance of targeted interventions for children recently initiating ART.

Before the transition to dolutegravir-based pediatric regimens, young children (ages ≤9 years) had the worst VL suppression results in Malawi and other countries in the region [9]. Despite improved efficacy and tolerability of pediatric dolutegravir formulations compared to older protease inhibitor regimens, viral suppression remains suboptimal across several African cohorts, with rates of 73–86% reported in Malawi, Nigeria, Cameroon, and Mozambique [21–23]. Since dolutegravir resistance remains rare among children with high viral load, [24], insufficient adherence likely explains most high VL cases in this population

An important adherence challenge identified in IDIs was children’s resistance to take daily medication. Studies from resource-limited settings, including from the East African region, suggest that caregivers should engage in open and age-appropriate discussions with children about the importance of taking medication regularly, using language and explanations that the child can understand [25]. Understanding the child’s perspective and addressing their concerns, such as fear of side effects or the taste of medication, can help alleviate resistance [26]. Monitoring medication consumption can ensure that children are taking their medication as prescribed [27], while teaching children swallowing techniques and providing them with positive reinforcement can make the process less daunting [28]. Regular follow-up visits and open communication channels between caregivers and HCWs can help identify and address adherence issues promptly, and peer support groups for caregivers can provide a platform for sharing experiences, learning from others, and receiving emotional support [29]. However, most of these interventions may be time consuming and require dedicated staff with appropriate expertise, which may challenge feasibility and reach in many settings in Malawi and other countries in the region.

Consistent with studies from low- and middle-income countries [30,31], we found that caregivers and HCWs see the pill burden for children as high and challenging for adherence. Strategies like dissolving medications, using more palatable medication formulations, and combining multiple medications into a single dose [32] could help simplify the pill regimens for children and improve adherence [32,33]. Future research should focus on developing and evaluating these strategies, as well as exploring novel approaches such as long-acting formulations and enhanced caregiver education and support [30,34–36].

Lack of food was major concern for caregivers, directly affecting medication adherence. Caregivers expressed that children experience more side effects when ART is taken on an empty stomach and that children are more likely to accept pills when given with food, although we could not find supportive literature for these observations. Some caregivers falsely believed that taking ART with food is a necessary requirement, a belief that may still stem from the older protease inhibitor-based pediatric formulation that needed to be taken with food. The impact of food insecurity on medication adherence has been widely reported [37,38], and our findings align with this literature. Nutritional support, currently not widely available in Malawi for non-malnourished children, and education about correct medication administration in relation to food are crucial for adherence and VL suppression in contexts with high rates of food insecurity [39,40].

Caregivers expressed concern that disclosing the HIV status to their child may lead to unintentional HIV status disclosure to other young children, thereby disclosing caregiver’s positive status in the community, with negative consequences for children and caregivers due to stigma. This highlights a complex social and emotional dilemma caregivers face in managing their child’s HIV status. Previous studies, including a systematic review, have shown the beneficial impact of HIV status disclosure on medication adherence of CWH [41], as proper disclosure helps them understanding their situation and motivates them to manage their own medication. Recent studies have also suggested that preparing children for treatment responsibility can start as early as 6 years of age [42], which is now supported in Malawi’s national guidelines (reference). However, the fear of caregivers for indirect disclosure of their own HIV status may hamper disclosing the HIV status to CWH. Understanding these concerns and addressing them through support programs for CWH and their caregivers may facilitate age-appropriate disclosure.

Lastly, caregivers in our study reported having trouble consistently attending scheduled clinic appointments resulting in IIT in the child. We found similar reasons for missed visits compared to those reported in studies of caregivers in Zambia and Kenya, including transportation costs, travel obligations, caregiver illness, competing life priorities, and conflicts with school hours [12,43]. Irregular clinic attendance often leads to missing medication refills, adherence counseling and monitoring of treatment response, all of which are crucial for maintaining VL suppression [44–46]. Implementing strategies such as appointment reminders via text messages or phone calls, offering special clinic days or extended hours for school-attending children, multi-month medication dispensing aligned with caregivers’ clinic appointments and innovative approaches to decentralizing HIV care may help minimizing missed clinic appointments [47–50].

While our study provides valuable insights into the factors associated with high VL and barriers to adherence in CWH, it is important to acknowledge its limitations. The qualitative data from caregivers and HCWs may be subject to recall and social desirability bias. As we relied on routinely collected data in the quantitative analysis, we could not include comprehensive sociodemographic data. Additionally, the study was conducted in a limited number of regions in Malawi, and the findings may not be generalizable to other contexts with different socio-economic and cultural characteristics. Our study excluded children with viral loads between 200–999 copies/mL, which limits understanding of causes of low-level viremia. Furthermore, the cross-sectional nature of the quantitative analysis precludes determining causal associations with high VL.

## Conclusion

We found that a number of behavioral, socio-economic and psychosocial factors were associated with high VL in CWH, with many factors related to dynamics in the community and family. Future interventions to improve viral load and health outcomes in CWH should include multi-level approaches, including age-appropriate disclosure, addressing food insecurity, and strategies to improve caregivers’ clinic attendance. Special attention should be given to children in their early years of treatment, when they are higher risk for high VL. The development of safe and effective long-acting ART for children could overcome many challenges to viral suppression and should be a priority for HIV therapeutics research.

## References

[1] Dow DE, Shayo AM, Cunningham CK, Reddy EA. Durability of antiretroviral therapy and predictors of virologic failure among perinatally HIV-infected children in Tanzania: a four-year follow-up. BMC Infect Dis. 2014;14:567. doi: 10.1186/s12879-014-0567-3 25373425 PMC4225040

[2] Aboud M, Kaplan R, Lombaard J, Zhang F, Hidalgo JA, Mamedova E, et al. Dolutegravir versus ritonavir-boosted lopinavir both with dual nucleoside reverse transcriptase inhibitor therapy in adults with HIV-1 infection in whom first-line therapy has failed (DAWNING): an open-label, non-inferiority, phase 3b trial. Lancet Infect Dis. 2019;19(3):253–64. doi: 10.1016/S1473-3099(19)30036-2 30732940

[3] Kanters S, Vitoria M, Zoratti M, Doherty M, Penazzato M, Rangaraj A, et al. Comparative efficacy, tolerability and safety of dolutegravir and efavirenz 400mg among antiretroviral therapies for first-line HIV treatment: A systematic literature review and network meta-analysis. EClinicalMedicine. 2020;28:100573. doi: 10.1016/j.eclinm.2020.100573 33294805 PMC7700905

[4] Paton NI, Musaazi J, Kityo C, Walimbwa S, Hoppe A, Balyegisawa A. Dolutegravir or darunavir in combination with zidovudine or tenofovir to treat HIV. N Engl J Med. 2021;385(4):330–41. 34289276 10.1056/NEJMoa2101609

[5] Venter WDF, Moorhouse M, Sokhela S, Fairlie L, Mashabane N, Masenya M, et al. Dolutegravir plus Two Different Prodrugs of Tenofovir to Treat HIV. N Engl J Med. 2019;381(9):803–15. doi: 10.1056/NEJMoa1902824 31339677

[6] Walmsley S, Baumgarten A, Berenguer J, Felizarta F, Florence E, Khuong-Josses MA, et al. Brief report: dolutegravir plus abacavir/lamivudine for the treatment of HIV-1 infection in antiretroviral therapy-naive patients: week 96 and week 144 results from the SINGLE randomized clinical trial. J Acquir Immune Defic Syndr. 2015;70(5):515–9. 26262777 10.1097/QAI.0000000000000790 PMC4645960

[7] Organization WH. Updated recommendations on first-line and second-line antiretroviral regimens and post-exposure prophylaxis and recommendations on early infant diagnosis of HIV: interim guidelines: supplement to the 2016 consolidated guidelines on the use of antiretrovirals; 2018, [cited 20 May 2024]. Available from: https://www.who.int/publications/i/item/WHO-CDS-HIV-18.51

[8] Townsend CL, O’Rourke J, Milanzi E, Collins IJ, Judd A, Castro H, et al. Effectiveness and safety of dolutegravir and raltegravir for treating children and adolescents living with HIV: a systematic review. J Int AIDS Soc. 2022;25(11):e25970. doi: 10.1002/jia2.25970 36377082 PMC9663860

[9] UNICEF. Understanding and Improving Viral Load Suppression in Children with HIV in Eastern and Southern Africa; 2021. Available from: https://www.unicef.org/esa/reports/understanding-and-improving-vls

[10] Malawi Ministry of Health. Malawi HIV Program Data Q4. In: Department of HIV&AIDS and Viral Hepatitis, editor. Lilongwe: Malawi Goverment; 2023.

[11] Blanco JL, Marcelin A-G, Katlama C, Martinez E. Dolutegravir resistance mutations: lessons from monotherapy studies. Curr Opin Infect Dis. 2018;31(3):237–45. doi: 10.1097/QCO.0000000000000453 29634660

[12] Haberer JE, Cook A, Walker AS, Ngambi M, Ferrier A, Mulenga V, et al. Excellent adherence to antiretrovirals in HIV+ Zambian children is compromised by disrupted routine, HIV nondisclosure, and paradoxical income effects. PLoS One. 2011;6(4):e18505. doi: 10.1371/journal.pone.0018505 21533031 PMC3080873

[13] Davies M-A, Boulle A, Fakir T, Nuttall J, Eley B. Adherence to antiretroviral therapy in young children in Cape Town, South Africa, measured by medication return and caregiver self-report: a prospective cohort study. BMC Pediatr. 2008;8:34. doi: 10.1186/1471-2431-8-34 18771599 PMC2533648

[14] Müller AD, Bode S, Myer L, Roux P, von Steinbüchel N. Electronic measurement of adherence to pediatric antiretroviral therapy in South Africa. Pediatr Infect Dis J. 2008;27(3):257–62. doi: 10.1097/INF.0b013e31815b1ad4 18277933

[15] Guest G, Bunce A, Johnson L. How Many Interviews Are Enough?. Field Methods. 2006;18(1):59–82. doi: 10.1177/1525822x05279903

[16] Court R, Leisegang R, Stewart A, Sunpath H, Murphy R, Winternheimer P, et al. Short term adherence tool predicts failure on second line protease inhibitor-based antiretroviral therapy: an observational cohort study. BMC Infect Dis. 2014;14:664. doi: 10.1186/s12879-014-0664-3 25472544 PMC4266950

[17] Kumar S, Quinn SC, Kim KH, Musa D, Hilyard KM, Freimuth VS. The social ecological model as a framework for determinants of 2009 H1N1 influenza vaccine uptake in the United States. Health Educ Behav. 2012;39(2):229–43. doi: 10.1177/1090198111415105 21984692 PMC3916095

[18] Fataha NVFA, Gaveta S, Sacarlal J, Rossetto EV, Baltazar CS, Kellogg TA. Characteristics associated with viral suppression among HIV-infected children aged 0-14 years in Mozambique, 2019. PLoS One. 2024;19(7):e0305380. doi: 10.1371/journal.pone.0305380 39024349 PMC11257270

[19] Lokpo S, Ofori-Attah P, Ameke L, Obirikorang C, Orish V, Kpene G. Viral suppression and its associated factors in HIV patients on highly active antiretroviral therapy (HAART): A retrospective study in the Ho municipality, Ghana. J Med Res. 2020;10(2):123–30.

[20] Berihun H, Bazie GW, Beyene A, Zewdie A, Kebede N. Viral suppression and associated factors among children tested for HIV viral load at Amhara Public Health Institute, Dessie Branch, Ethiopia: a cross-sectional study. BMJ Open. 2023;13(1):e068792. doi: 10.1136/bmjopen-2022-068792 36720566 PMC9890760

[21] Mugo C, Zubayr B, Ezeokafor N, Oyawola B, Ekele DO, Madueke L, et al. Effect of Dolutegravir and Multimonth Dispensing on Viral Suppression Among Children With HIV. J Acquir Immune Defic Syndr. 2023;93(3):229–36. doi: 10.1097/QAI.0000000000003190 36943698 PMC10256312

[22] Fokam J, Nka AD, Mamgue Dzukam FY, Efakika Gabisa J, Bouba Y, Tommo Tchouaket MC, et al. Viral suppression in the era of transition to dolutegravir-based therapy in Cameroon: Children at high risk of virological failure due to the lowly transition in pediatrics. Medicine (Baltimore). 2023;102(20):e33737. doi: 10.1097/MD.0000000000033737 37335723 PMC10194733

[23] Gill MM, Herrera N, Guilaze R, Mussa A, Dengo N, Nhangave A, et al. Virologic Outcomes and ARV Switch Profiles 2 Years After National Rollout of Dolutegravir to Children Less Than 15 Years in Southern Mozambique. Pediatr Infect Dis J. 2023;42(10):893–8. doi: 10.1097/INF.0000000000004037 37409808

[24] Bello G, Pals SBB. Emerging dolutegravir resistance among children being investigated for treatment failure in Malawi. Denver: Conference on Retroviruses and Opportunistic Infections; 2024.

[25] Fetzer BC, Mupenda B, Lusiama J, Kitetele F, Golin C, Behets F. Barriers to and facilitators of adherence to pediatric antiretroviral therapy in a sub-Saharan setting: insights from a qualitative study. AIDS Patient Care STDS. 2011;25(10):611–21. doi: 10.1089/apc.2011.0083 21823909 PMC4530354

[26] Kunapareddy CJ, Nyandiko W, Inui T, Ayaya S, Marrero DG, Vreeman R. A qualitative assessment of barriers to antiretroviral therapy adherence among adolescents in Western Kenya. J HIV/AIDS Soc Ser. 2014;13(4):383–401. doi: 10.1080/15381501.2012.754392 PMC5374741 28367106

[27] Vreeman RC, Ayaya SO, Musick BS, Yiannoutsos CT, Cohen CR, Nash D, et al. Adherence to antiretroviral therapy in a clinical cohort of HIV-infected children in East Africa. PLoS One. 2018;13(2):e0191848. doi: 10.1371/journal.pone.0191848 29466385 PMC5842873

[28] Garvie PA, Lensing S, Rai SN. Efficacy of a pill-swallowing training intervention to improve antiretroviral medication adherence in pediatric patients with HIV/AIDS. Pediatrics. 2007;119(4):e893-9. doi: 10.1542/peds.2006-1488 17353298

[29] Funck-Brentano I, Dalban C, Veber F, Quartier P, Hefez S, Costagliola D, et al. Evaluation of a peer support group therapy for HIV-infected adolescents. AIDS. 2005;19(14):1501–8. doi: 10.1097/01.aids.0000183124.86335.0a 16135904

[30] Mistry P, Batchelor H, SPaeDD-UK Project (Smart Paediatric Drug Development - UK). Evidence of acceptability of oral paediatric medicines: a review. J Pharm Pharmacol. 2017;69(4):361–76. doi: 10.1111/jphp.12610 27524471

[31] Nasuuna E, Kigozi J, Muwanguzi PA, Babirye J, Kiwala L, Muganzi A, et al. Challenges faced by caregivers of virally non-suppressed children on the intensive adherence counselling program in Uganda: a qualitative study. BMC Health Serv Res. 2019;19(1):150. doi: 10.1186/s12913-019-3963-y 30845951 PMC6407183

[32] Bagenda A, Barlow-Mosha L, Bagenda D, Sakwa R, Fowler MG, Musoke PM. Adherence to tablet and liquid formulations of antiretroviral medication for paediatric HIV treatment at an urban clinic in Uganda. Ann Trop Paediatr. 2011;31(3):235–45. doi: 10.1179/1465328111Y.0000000025 21781419

[33] Nahirya-Ntege P, Cook A, Vhembo T, Opilo W, Namuddu R, Katuramu R, et al. Young HIV-infected children and their adult caregivers prefer tablets to syrup antiretroviral medications in Africa. PLoS One. 2012;7(5):e36186. doi: 10.1371/journal.pone.0036186 22567139 PMC3342167

[34] Walsh J, Cram A, Woertz K, Breitkreutz J, Winzenburg G, Turner R, et al. Playing hide and seek with poorly tasting paediatric medicines: do not forget the excipients. Adv Drug Deliv Rev. 2014;73:14–33. doi: 10.1016/j.addr.2014.02.012 24614069

[35] Schlatter AF, Deathe AR, Vreeman RC. The Need for Pediatric Formulations to Treat Children with HIV. AIDS Res Treat. 2016;2016:1654938. doi: 10.1155/2016/1654938 27413548 PMC4927993

[36] Network I. Phase I/II Study of the Safety, Acceptability, Tolerability, and Pharmacokinetics of Oral and Long-Acting Injectable Cabotegravir and Long-Acting Injectable Rilpivirine in Virologically Suppressed HIV-Infected Children and Adolescents accessed on 10 September 2024. [Trial protocol]. In Press; 2023.

[37] Nagata JM, Magerenge RO, Young SL, Oguta JO, Weiser SD, Cohen CR. Social determinants, lived experiences, and consequences of household food insecurity among persons living with HIV/AIDS on the shore of Lake Victoria, Kenya. AIDS Care. 2012;24(6):728–36. doi: 10.1080/09540121.2011.630358 22150119 PMC4418445

[38] Young S, Wheeler AC, McCoy SI, Weiser SD. A review of the role of food insecurity in adherence to care and treatment among adult and pediatric populations living with HIV and AIDS. AIDS Behav. 2014;18(Suppl 5):S505–15. doi: 10.1007/s10461-013-0547-4 23842717 PMC3888651

[39] Cantrell RA, Sinkala M, Megazinni K, Lawson-Marriott S, Washington S, Chi BH, et al. A pilot study of food supplementation to improve adherence to antiretroviral therapy among food-insecure adults in Lusaka, Zambia. J Acquir Immune Defic Syndr. 2008;49(2):190–5. doi: 10.1097/QAI.0b013e31818455d2 18769349 PMC3847664

[40] Benzekri NA, Sambou JF, Ndong S, Diallo MB, Tamba IT, Faye D, et al. The impact of food insecurity on HIV outcomes in Senegal, West Africa: a prospective longitudinal study. BMC Public Health. 2021;21(1):451. doi: 10.1186/s12889-021-10444-1 33676463 PMC7936446

[41] Vreeman RC, Gramelspacher AM, Gisore PO, Scanlon ML, Nyandiko WM. Disclosure of HIV status to children in resource-limited settings: a systematic review. J Int AIDS Soc. 2013;16(1):18466. doi: 10.7448/IAS.16.1.18466 23714198 PMC3665848

[42] Vaz LM, Eng E, Maman S, Tshikandu T, Behets F. Telling children they have HIV: lessons learned from findings of a qualitative study in sub-Saharan Africa. AIDS Patient Care STDs. 2010;24(4):247–56. 20397899 10.1089/apc.2009.0217 PMC2864057

[43] Vreeman RC, Nyandiko WM, Ayaya SO, Walumbe EG, Marrero DG, Inui TS. Factors sustaining pediatric adherence to antiretroviral therapy in western Kenya. Qual Health Res. 2009;19(12):1716–29. doi: 10.1177/1049732309353047 19949221

[44] Mugavero MJ, Lin H-Y, Willig JH, Westfall AO, Ulett KB, Routman JS, et al. Missed visits and mortality among patients establishing initial outpatient HIV treatment. Clin Infect Dis. 2009;48(2):248–56. doi: 10.1086/595705 19072715 PMC2737584

[45] Rastegar DA, Fingerhood MI, Jasinski DR. Highly active antiretroviral therapy outcomes in a primary care clinic. AIDS Care. 2003;15(2):231–7. doi: 10.1080/0954012031000068371 12856344

[46] Nasuuna E, Kigozi J, Muganzi A, Sewankambo N, Nakanjako D. Short report: knowledge and perceptions of health workers that strengthen adherence for paediatric and adolescent clients on the intensive adherence counselling program in Kampala, Uganda: a qualitative study. Afr Health Sci. 2021;21(Suppl):25–8. doi: 10.4314/ahs.v21i1.5S 34447420 PMC8367312

[47] Kunutsor S, Walley J, Katabira E, Muchuro S, Balidawa H, Namagala E, et al. Clinic Attendance for Medication Refills and Medication Adherence amongst an Antiretroviral Treatment Cohort in Uganda: A Prospective Study. AIDS Res Treat. 2010;2010:872396. doi: 10.1155/2010/872396 21490907 PMC3065731

[48] Pop-Eleches C, Thirumurthy H, Habyarimana JP, Zivin JG, Goldstein MP, de Walque D, et al. Mobile phone technologies improve adherence to antiretroviral treatment in a resource-limited setting: a randomized controlled trial of text message reminders. AIDS. 2011;25(6):825–34. doi: 10.1097/QAD.0b013e32834380c1 21252632 PMC3718389

[49] Grimsrud A, Lesosky M, Kalombo C, Bekker L-G, Myer L. Implementation and operational research: community-based adherence clubs for the management of stable antiretroviral therapy patients in Cape Town, South Africa: A cohort study. J Acquir Immune Defic Syndr. 2016;71(1):e16-23. doi: 10.1097/QAI.0000000000000863 26473798

[50] Songo J, Whitehead HS, Nichols BE, Makwaya A, Njala J, Phiri S, et al. Provider-led community antiretroviral therapy distribution in Malawi: Retrospective cohort study of retention, viral load suppression and costs. PLOS Glob Public Health. 2023;3(9):e0002081. doi: 10.1371/journal.pgph.0002081 37768889 PMC10538660

